# Multiple Xenosteroid Pollutants Biomarker Changes in Cultured Nile Tilapia Using Wastewater Effluents as Their Primary Water Source

**DOI:** 10.3390/ani10091475

**Published:** 2020-08-22

**Authors:** Eman Zahran, Mohammed Elmetwally, Walaa Awadin, Mansour El-Matbouli

**Affiliations:** 1 Clinical Division of Fish Medicine, University of Veterinary Medicine, Veterinärplatz 1, 1210 Vienna, Austria; 2 Department of Internal Medicine, Infectious and Fish Diseases, Faculty of Veterinary Medicine, Mansoura University, Mansoura 35516, Egypt; 3 Department of Theriogenology, Faculty of Veterinary Medicine, Mansoura University, Mansoura 35516, Egypt; metwally@mans.edu.eg; 4 Departments of Pathology, Faculty of Veterinary Medicine, Mansoura University, Mansoura 35516, Egypt; walafekryawadine@mans.edu.eg

**Keywords:** EDCs, fish, Lake Manzala, hormonal disturbances, reproductive gene dysfunction, histopathology

## Abstract

**Simple Summary:**

Estrogenic endocrine disruptive chemicals (E-EDCs) are important types of pollutants in fish farms worldwide and a globally concerned problem. In this study, Nile tilapia fish farms receiving wastewater effluents in Egypt were selected as highly, moderately polluted fish farms; besides, a putative control site was deemed low in contamination. Levels of E-EDCs (natural and synthetic steroids, and industrial phenolic compound/bisphenol A (BPA)) was recorded in farm water, and fish tissues at all sites under consideration, mainly, lower levels of testosterone, progesterone, zeranol, and 17β-estradiol were detected compared to the higher level of BPA. Moreover, the effects of these pollutants on fish biometric, reproductive genes, and hormonal biomarkers was evaluated along with the observed associated histopathological alterations. Our findings revealed the detection of some steroidal compounds with a higher level of the BPA. All analyzed biomarkers were reduced to a great extent in the highly polluted sites compared to others, and the histopathological alterations observed were supportive of other measurements. These observations warrant strict monitoring of aquatic pollution sources and the development of strategic plans to control aquaculture pollution.

**Abstract:**

This study was undertaken to screen levels of xenosteroids (estrogenic endocrine disrupting chemicals/E-EDCs) in Nile tilapia (*Oreochromis niloticus*) fish farms subjected to water fill from the drain at three sites S1 (highly polluted), S2 (moderately polluted), and a putative reference site (RS). Biometric, hormonal, gene expression, and histopathological analysis were investigated. Testosterone, progesterone, and zeranol residues were detected at (0.12–3.44 µg/L) in water samples of different sites. Bisphenol-A (BPA) exhibited a very high concentration (6.5 µg/mL) in water samples from S1. Testosterone, 17β-estradiol residues were detected in fish tissues from all sites at (0.16–3.8 µg/Kg) and (1.05–5.01 µg/Kg), respectively. BPA residues were detected at a very high concentration in the liver and muscle of fish collected from S1 at higher levels of 25.9 and 48.07 µg/Kg, respectively. The detected E-EDCs, at different sites, particularly BPA, reduced the somatic and testicular growth among sites and oversampling time points. Meanwhile, hepatosomatic index (HSI) was significantly increased in S1 compared to S2. All analyzed genes estrogen receptor-type I (*er-I*, *er-ɑ*) and II (er-*II, er-ß1*), polypeptide 1a (*cyp19a1*), SRY-box containing gene 9 (*sox9*), and vitellogenin (*vtg*) and gonadotropin hormones (luteinizing hormone (LH), follicle-stimulating hormone (FSH)), testosterone, 17β-estradiol, and anti-Mullerian hormone (AMH) were significantly expressed at S1 compared to other sites. Histopathology was more evident in S1 than other sites. These findings warrant immediate strategies development to control aquatic pollution and maintain fish welfare and aquaculture sustainability.

## 1. Introduction

The aquaculture industry in Egypt is a fast-growing sector contributing about 77% of the total national fish production [1,2,3]. Reproductive performance is the crucial feature in interpreting the population dynamics of fish that have implications for fish sustainability [4]. Nile tilapia (*Oreochromis niloticus*) is a worldwide highly adaptable cultured species [5], reproduce continuously throughout the year, and their gonads are ripe all year round; however, the breeding season peaks from April to August depending on the temperature, solar radiation, or rainy season and rise in water level [6]. The increased demand for fish production as a vital source of animal protein, mainly in urban and rural areas, has led to over expansion of aquaculture [7,8,9]. However, serious constraints are emerging as a result of aquaculture expansion. Notably, to reserve high-quality water for human and agricultural use, Egyptian legislation only allows aquaculture facilities to make use of water from drains, including water that had been released from wastewater plants or had been used for agricultural purposes. Several studies have reported that pollutants can elicit reproductive impairment in fish [10]. In this context, estrogenic endocrine disrupting chemicals (E-EDCs) pollution is considered one of the most critical constraints of the aquatic ecosystem. E-EDCs are compound with an estrogen-like activity that can interfere with the hormonal balance required for homeostasis and regulation of the developmental process [11]. Different types of contaminants, including xenosteroid, find their way to rivers, lakes, and aquatic ecosystems through anthropogenic sources and agriculture runoff [12]. Due to their aquatic habitat, fish are in intimate contact with pollutants released from industry, agriculture, and domestic sources [13]. Therefore, fish are considered a good indicator of aquatic ecosystem health [14]. E-EDCs have recently attracted increased scrutiny, not only for their toxicological hazards in the ecosystem but also for its impact on human beings [15]. E-EDCs are either naturally produced, such as estrone (E1), 17β-estradiol (E2), and estriol (E3), or synthetic, such as contraceptive 17α-ethynylestradiol (EE2) [16]. E1, E2, E3, and EE2 potentially induce their effect in concentrations as low as nanogram/liter level. They are mainly responsible for in vitro estrogenic activity [17,18] and are detected in domestic wastewater and wastewater treatment plant (WWTP) effluents [19,20,21], discharged finally into lakes or rivers [22]. However, most of the sulfate or glucuronide conjugated form of excreted hormones are biologically inactive; however, they can exert their adverse effects after being transformed into biologically active compounds by the action of bacteria [23].

Additionally, E-EDCs as nonylphenol (NP), 4-octylphenol (OP), and bisphenol A (BPA) are released from domestic, industrial, and WWTP effluents, estrogenic at microgram/liter levels and lead to endocrine disruption in aquatic organisms [24,25,26]. BPA is incorporated in various industrial, household, and agricultural applications [27] that raise public health issues [28]. BPA concentrations have been detected in the range of 0.01 to 44.7 μg/L in river water [29] and in fish muscles <0.18–2.60 μg/kg [30,31].

E-EDCs in fish are associated with severe negative impacts on the biological system and normal body functions, including reducing their resistance against diseases [32]. Xenosteroids could elicit promotion and/or suppression of estrogenic response, induce reproductive dysfunction, disrupt gene expression, affect gonadal development, and lower or abolish fecundity [33]. These negative impacts result in community depletion, species composition changes, or even species extinction [15,34].

E-EDCs can lead to disruption of the sex steroid hormone system through binding to the steroid hormone receptor or affecting its release, pathways, and function [35]. Reproductive-related gene expression analysis progressively enhanced a better understanding of the mechanistic action of these chemicals and give the ability to assess the different mode of actions for various compounds as well [36].

Several studies have reported different screened levels of EDCs and their fate in WWTPs. A few studies have investigated E-EDCs in aquaculture, focusing on either experimental exposure and bioaccumulation or natural exposure and bioaccumulation in the tissue of fish exposed to steroids in polluted waters [37]. However, no studies have yet evaluated different aspects of the E-EDCs effects on fish. To the best of our knowledge, our study is the first comprehensive study to investigate the effects of environmental levels of steroidal and phenolic xenosteroids, occurrence, bioaccumulation, biometric, hormonal biomarkers, gene expression disruption; and histopathological alterations in Nile tilapia fish farms located close to the Bahr El-Baqar drain of Lake Manzala (Egypt).

The objectives of the present study were to (1) determine the levels of occurrence and bioaccumulation of E-EDCs in aquaculture farms close to Bhar El-Baqar drain of Lake Manzala (Egypt), (2) analyze the biometric response to E-EDCs analyzed levels and, (3) elucidate effects of E-EDCs on hormonal biomarkers, gene expression; and histopathological alterations induced.

## 2. Materials and Methods

### 2.1. Study Area 

The study areas consist of fish farms nearby and receiving water from the Bahr El-Baqar drain (Latitude, 31°1′40″, Longitude, 32°09′24″). The Bahr El-Baqar drain is one of the most important drainage ditches by which vast wastewater is emptied into Lake Manzala, the largest one of the northern Nile delta lakes in Egypt [38,39]. The Bahr El-Baqar drainage carries municipal and industrial waste untreated water from east of Cairo [40]. The drainage serves about 2252 km^2^ of the agricultural area that forms about 25% of the total inflow [41].

**Figure 1:**
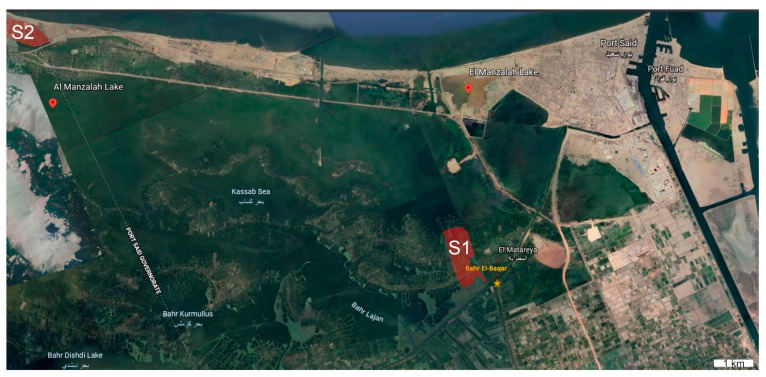
The study area of Manzala lagoon in the northeastern Nile delta of Egypt (Bahr El-Baqar drain, sampling sites in red marks). S1: Site 1; S2: Site 2.

The three primary sources of pollutants in the Bahr El-Baqar region are the industrial activities, wastewater discharges, and domestic discharges received from rural areas around the Bahr El-Baqar drain [42]. The Bahr El-Baqar drain is used as the water source for fish farms located in its proximity [39,43]. Semi-intensive ponds are the most common aquaculture practiced in this area. In the present study, two typical pond aquaculture fish farms were used: Site 1 (S1) corresponds to the closest point to the drainage about 1 km, the fish farm about 6.3 Hectare (ha), and mortality rates were about 35 %. Site 2 (S2) covers a fish farm that is far from the drainage source, about 35 km. The fish farm is about 4.6 ha, and mortality rates were about 5%. A putative control (reference site, RS) site was chosen as their water source originated from the underground water source and was deemed low in contaminants (Figure 1).

### 2.2. Samples Collection and Processing

A total of 180 male monosex adult Nile tilapia (*O. niloticus*) were collected from all sampling sites between August and October 2017 (*n* = 20 fish/site/month). Fish were transported alive in aerated coolers containing water from the collection site until processed at the Fish Diseases and Management laboratory (FDM), Faculty of Veterinary Medicine, Mansoura University. Fish were euthanized with an overdose of buffered MS-222 (one fish at the time) in a separate container (200 mg/L MS-222 + 400 mg/L sodium bicarbonate). Each fish was weighed (to the nearest gram) and measured (to the nearest centimeter). Fish then were dissected with a clean scalpel blade to separate the tissues. The liver and testes were weighed per each fish to calculate the testicular somatic index (TSI) and hepatosomatic index (HSI). Fish samples were pooled (10 individual/site) for gene expression, hormones analysis. The liver and testes were divided into two and three portions, respectively. The first portion of the liver and testes, in addition to the muscle (one pool/tissue), kept separately and stored at −20 °C until xenosteroid analysis. The second portion of the liver and testes were kept in RNAlater^®^ (Sigma, Deisenhofen, Germany) and stored at −20 °C for gene expression analysis. The third portion of the testes (60 fish/site/study period) were fixed in 10% neutral buffer formalin for histopathological analysis.

A total of 18 single, discrete water samples were collected in an amber glass bottle (1 L) at all of the fish sampling locations on the same day of each fish collection and transported in coolers to the FDM laboratory. Water samples were then acidified to pH 3 by adding nitric acid (65%) within 4 h of the collection to dissolve metals and prevent oxidation, kept on ice, and stored at 4 °C until analysis. 

Fish biometric and organosomatic measurements, including weight (Wt), length (cm), HSI, and testicular-somatic index (TSI), were measured in each 20 fish/site/month. HSI = Wt of liver (g)/body wt (g) × 100; TSI = Wt of testes (g)/body wt (g) × 100. All fish capture, handling, and euthanasia procedures complied with the Animal Care and Use Guidelines at Mansoura University, Egypt and approved by the local Administrative Panel on Laboratory Animal Care Committee.

### 2.3. Extraction and Quantification of Estrogenic Endocrine Disrupting Chemicals (E-EDCs) in Fish Tissues and Water Samples

Natural, synthetic steroid hormones and veterinary pharmaceuticals analysis were performed for all water samples and fish tissues (testes, muscle, and liver) in a certified Central Laboratory of Residual Analysis of Pesticides and Heavy Metals (QCAP); FINAS (Finnish Accredited Service; T219 (EN ISO/IEC 17025). Ultrasonic solvent extraction and liquid chromatography–tandem mass spectrometry (LC-MSMS, Thermo Fisher Scientific, San Jose, CA, USA), was used according to Zavala et al. [44], and the concentrations of estrone (E1), 17β-estradiol (E2), hexestrol, diethylstilbestrol, dienestrol, testosterone, progesterone, and zeranol were measured. The measurement uncertainty expressed as expanded uncertainty (at 95% confidence level) was within the range ± 0.33%. The limit of quantification (LOQ) was 0.1 µg/kg. BPA extraction and analysis in fish tissues (muscle and liver) and water samples were carried out at the Central Laboratories Network, National Research Centre, Cairo, Egypt, according to Xiao et al. [45]. Briefly, HPLC analysis was carried out using an Agilent 1260 series. The separation was carried out using a C18 column (4.6 mm × 250 mm i.d., 5 μm). The mobile phase consisted of 10 mM monopotassium phosphate and acetonitrile (60:40) at a flow rate of 1 mL/min and run time of 12 min. The multi-wavelength detector was monitored at 250 nm. The injection volume was 30 μL for each of the sample solutions. The column temperature was maintained at 40 °C. 

### 2.4. Gene Expression Analysis

Gene expression analyses were performed by RT-qPCR. Total RNA was extracted from liver and testes samples (30 mg); using an RNeasy Mini Kit (Qiagen, Hilden, Germany) according to the manufacturer’s instructions. The purity and quantity of the resulting RNA were confirmed spectrophotometrically by measuring the optical density at 260/280 nm. From total RNA, 1 µg was reverse transcribed using an iSCRIPT^®^ (Bio-Rad, München, Germany) cDNA synthesis kit per the user’s manual and stored at −20 °C before qualitative and quantitative PCR analysis. A gradient PCR was performed to determine the best annealing temperature for each primer (Table A1). All qPCR reactions were carried out in duplicates using a CFX96 Touch Real-Time PCR detection system (BIO-RAD, München, Germany) with an SsoAdvanced Universal SYBR Green Supermix (Bio-Rad, München, Germany) to quantify gene expression levels in the testes and liver samples. RT-qPCR in a final volume of 20 μL contained 5 μL of 1:4-fold diluted cDNA, 0.01 μM of each primer, 1× SYBR Green Supermix (Bio-Rad, München, Germany), and sterile distilled water. After 5 min of cDNA denaturation at 95 °C, 35 cycles were performed at 95 °C for 30 s, annealing for 30 s at the temperatures indicated in Table A1, and extension at 72 °C for 30 s. After the cycling protocol, the melting curves were obtained to assess the specificity. The threshold cycle (Ct) was determined for each reaction for the quantification of the PCR results. The Ct values for the gene of interest were normalized to the endogenous control gene, β-actin, by using the 2^−∆∆Ct^ method [46]. The normalized values obtained were used to calculate the expression levels relative to β-actin. Sources of transcript-specific primers (Table A1) were as follow: β-actin (this study), (estrogen receptor-type I (*er-I, er-ɑ*) and II (*er-II, er-ß1*), cytochrome P450 aromatase, family 19, subfamily A, polypeptide 1a (*cyp19a1*) [35]; SRY-box containing gene 9 (*sox9*) [47], and vitellogenin (*vtg*)) [48].

### 2.5. Hormones Analysis

Blood samples were collected from the caudal vein using a 2 mL syringe and transferred into a plain centrifuge tube, allowed to clot at room temperature for 20 min, then left at 4 °C for 4 h. The clotted samples were then centrifuged at 1198× *g* for 10 min, and the sera were collected and stored at −20 °C for later analysis. Serum luteinizing hormone (LH), follicle-stimulating hormone (FSH), 17β-estradiol (E2), and testosterone (T) were analyzed using a competitive chemiluminescent enzyme immunoassay (Immulite 1000, Siemens, Los Angeles, CA, USA). Duplicate samples were assayed at the same time, in a single run with a single lot number of reagents and consumables employed by a single operator, with intra-assay coefficients of variation for all variables less than 5%. Anti-Mullerian hormone (AMH) hormone levels were assayed using an inhouse double antibody enzyme-linked immunosorbent assay (ELISA), with intra- and inter-assay coefficient of variation of <5% and <10%, respectively [49].

### 2.6. Tissue Histopathology

Testes were collected from the captured fish, fixed in 10% neutral buffered formalin for 24 h, dehydrated through a graded series of ethanol, cleared with xylene solutions, and embedded in a block using melted paraffin. The paraffin blocks were sectioned at 6 µm thickness using a rotary microtome and stained with hematoxylin and eosin (H&E) [50]. 

The stained sections were examined with a light microscope (OLYMPUS CX41, Olympus Corporation, Tokyo, Japan) and photographed using a digital camera. A semi-quantitative histological assessment protocol based on the scoring scheme developed by Zimmerli et al. [51] was applied with some modifications to quantify histological alterations observed in the testes. Testicular alterations were assessed according to six reaction patterns: circulatory disturbances (CD), retrogressive changes (RC), progressive change (PC), inflammation (I), neoplasm (N), and intersex (IS). Every alteration was assessed using a score ranging from 0 to 6, depending on the degree and extent of the alteration: (0) unchanged; (2) mild occurrence; (4) moderate occurrence; and (6) severe occurrence (diffuse lesion). Intermediate values were also considered. The sum of these indices yielded the total testicular index (TI) in each fish. These indices indicate the extent and intensity of histological alteration in the respective tissue and convert qualitative observations on alterations in tissue morphology into a quantitative value. The minimum TI was 0, and the maximum TI was 36. These index values were used to compare the severity of the occurrence of testicular histological alterations between fish from RS, S1, and S2.

- TI <10: normal/healthy structure; tissue architecture and histology were well developed and showed no impairments or pathological changes.
- TI 11–20: slight modifications of normal tissue architecture and morphology (e.g., change in cell size) were present.
- TI 21–30: moderate modifications of normal tissue architecture and morphology were present.
- TI 31–36: pronounced modifications of normal tissue architecture and morphology were present.

### 2.7. Statistical Analysis

Data normality and homoscedasticity were analyzed by the Kolmogoro–-Smirnov and Levene tests, respectively. Biometric and organosomatic parameters analyses were performed using the GraphPad prism software for Windows version 5.01(GRAPH PAD software Inc, CA, USA). A two-way ANOVA followed by Bonferroni’s multiple comparisons test was used to examine the possible effect of sites, sampling time points, and interaction on the biometric and organosomatic parameters. Gene expression and hormonal parameter analyses were performed using the SPSS software for Windows version 24.0 (SPSS, Chicago, IL, USA). A one-way analysis of variance (ANOVA), followed by Duncan’s multiple range test was used to examine the possible effect of sites on gene expression and hormonal parameters. The effects with a probability of *p* < 0.05 were considered significant. Additionally, an independent *t*-test was performed to compare FSH and LH levels at each site, and effects with a probability of *p* < 0.05 are statistically significant. Gene expression and hormonal parameters figures were graphically presented using Microsoft Excel 2016 (Redmond, WA, USA). Histopathological data were analyzed using the GraphPad prism software for Windows version 5.01 (GRAPH PAD software Inc, CA, USA). Kruskal–Wallis and Dunn’s tests were used to compare mean values between sites, and effects with a probability of *p < 0.05* are statistically significant.

## 3. Results

### 3.1. Estrogenic Endocrine Disrupting Chemicals (E-EDCs) Analysis in Fish Tissues and Water 

The levels of all xenosteroids analyzed in water and fish tissues from different sites are presented in Table 1. Testosterone was detected in water sampled from RS (0.12 µg/L) and S1 (0.2 µg/ L). Progesterone was higher at RS (0.21 µg/L) than S1 (0.13 µg/L) and not detected in S2. Zeranol was higher in S2 (3.44 µg/L) compared to RS (0.51 µg/L) and not detected in S1. Other xenosteroids tested were below detectable levels at all sampled sites. Phenolic EDCs, BPA, was detected at S1 at levels of 6.5 µg/mL. Bioaccumulation of testosterone, 17β-estradiol, and BPA were detected in fish tissues. Liver testosterone residue was higher compared to muscle and testes. However, testicular testosterone residues at S1 was the highest at the level of 6 µg/kg. 17β-estradiol residues in the testes were the highest at S2 (5.01 µg/kg). Additionally, 17β-estradiol was also detected in muscle tissue of fish collected from S1. Interestingly, elevated levels of BPA residues were found in the muscle and liver of fish collected from S1 (25.9 µg/kg and 48.07 µg/kg, respectively).

### 3.2. Fish Biometric and Organosomatic Measurements

**Figure 2:**
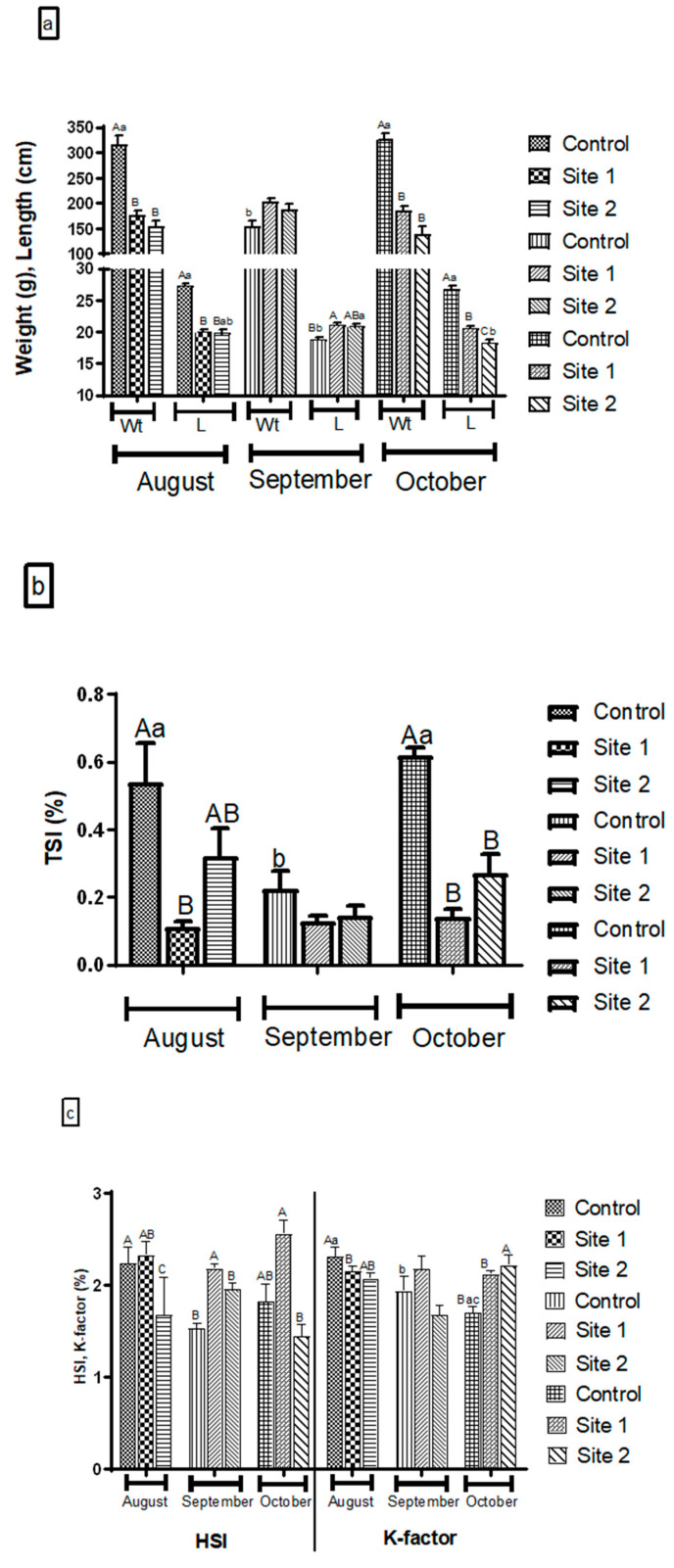
(**a**) Weight (Wt) and Length (L), (**b**) testicular somatic index (TSI), (**c**) hepatosomatic index (his), and K-factor of Nile tilapia collected from different sampling sites. Data are presented as the mean of 20 fish ± standard error of mean (SEM). Values with a different uppercase letter superscript indicate a significant difference between sites at the same sampling time. Values with different lowercase letter superscript are significantly different between sampling time points within the same site (*p* < 0.05, two-way ANOVA).

Fish weight and length were in line, where a significant increase (*p* < 0.001) was evident in fish sampled from RS during August and October months compared to September months, with no significant changes between the former. Fish length from S2 was significantly increased during September compared to October months. Concerning changes among sites at each sampling time, fish weight and length data were consistent during August and October months, where a significant decrease (*p* < 0.001) in S1 and S2 was noted compared to RS, besides, a significant decrease (*p* < 0.01) in fish length only at S2 compared to S1 during October month. Meanwhile, during September, the fish length was significantly increased (*p* < 0.05) at S1 compared to RS with no statistical changes to S2. Times*sites interaction (two-way ANOVA, *p* < 0.05) had a significant effect on fish weight and length (Figure 2a). 

TSI result was also consistent with the weight and length patter, where a significant increase was evident during August (*p* < 0.01) and October (*p* < 0.001) months compared to September month. Besides, a significant decrease (*p* < 0.001) in fish was captured from S1 compared to RS during August and October. Additionally, TSI of fish captured from S2 was significantly reduced (*p* < 0.01) compared to RS during October month, but with no significant differences compared to both S1 and RS during August month (Figure 2b). 

HSI showed a significant increase ((*p* < 0.01), (*p* < 0.001), (*p* < 0.05)) in S1 compared to S2 and during August, September, and October months, respectively, while no statistical differences compared to RS, excluding September months. K-factor showed a significant decrease in S1 compared to RS during August, while it demonstrated a significant increase in S2 compared to RS during October months. There was a significant decrease and increase in HSI of fish from RS during September compared to August and October months, respectively. Times*sites interaction (two-way ANOVA, *p* < 0.05) had a significant effect on both HSI and K-factor values (Figure 2c).

### 3.3. Endocrine Gene Expression Analysis in Testes and Liver Tissues

**Figure 3:**
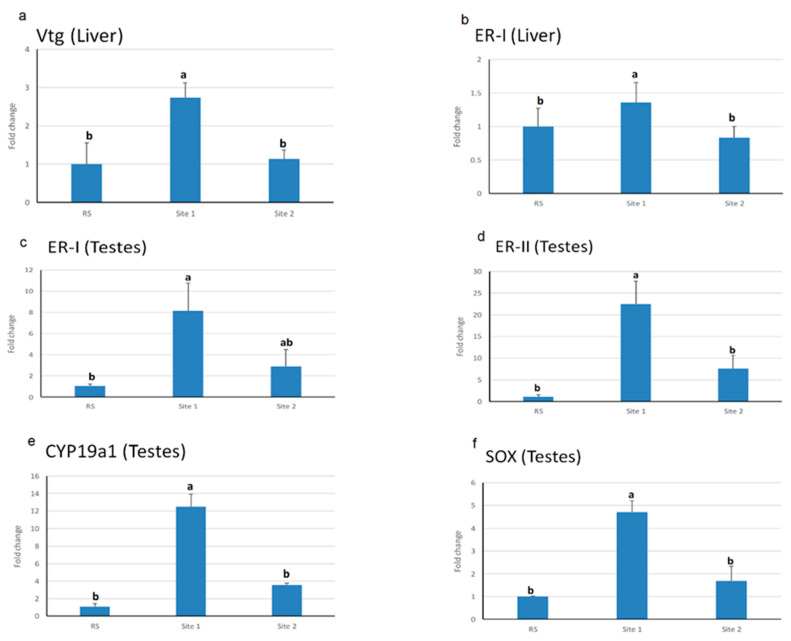
Vitellogenin (*vtg*) (**a**), estrogen receptor-type I (*er-I*) (**b**) mRNA levels in the liver; and *er*-I (**c**), *er–II* (**d**), polypeptide 1a (*cyp19a1*) (**e**), SRY-box containing gene 9 (*sox9*) (**f**) mRNA levels in testes relative to β-actin mRNA levels analyzed by qRT-PCR in Nile tilapia collected from different sampling sites. Data are presented as the mean of 10 fish ± standard error of mean (SEM). Values with a different letter superscript indicate a significant difference between sites (*p* < 0.05, one-way ANOVA). RS = control.

Testes *er-type II, cyp19a1,* and *sox9* mRNA levels were significantly upregulated in S1 (*p* < 0.001) compared to S2 and RS, while there is no statistical change between the latter. However, *er-type I* mRNA level in testes was significantly upregulated in S1 compared only to RS (*p* < 0.001), while there were no significant changes in S2 compared to S1and RS. Similarly, *vtg* and *er–type I* mRNA levels in the liver exhibited significant upregulation in S1 (*p* < 0.05) compared to S2 and RS with no statistical changes between the latter (Figure 3).

### 3.4. Hormones Analysis

**Figure 4:**
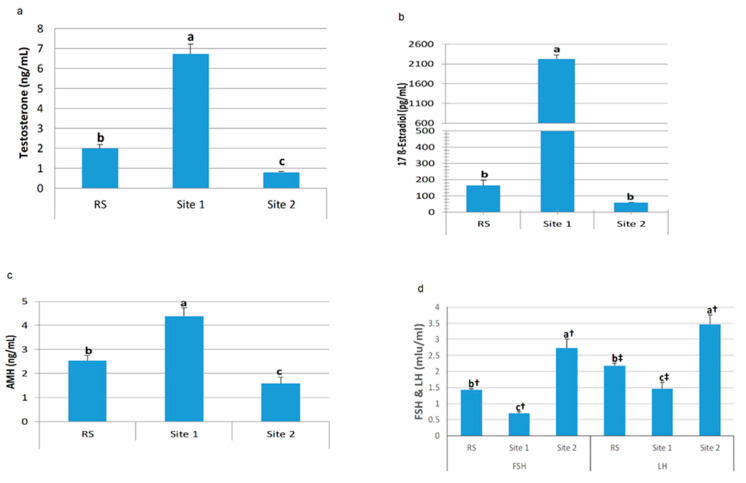
Testosterone (**a**), 17β-estradiol (E2) (**b**), anti-Mullerian hormone (AMH) (**c**), follicle-stimulating hormone (FSH) and luteinizing hormone (LH) (**d**) serum hormonal analysis of Nile tilapia collected from different sampling sites. Data are presented as the mean of 10 fish ± standard error of mean (SEM). Values with a different letter superscript indicate a significant difference between sites (*p < 0.05*, one-way ANOVA). Values with a different dagger superscript indicate a significant difference between FSH and LH levels in the same site (*p < 0.05*, independent *t*-test). RS = control.

LH and FSH levels were in a similar pattern where; there were significant differences among all sites, being S2 showing the highest level. However, there was a significant increase in LH levels compared to the FSH levels at S1, with no significant changes at S2. Testosterone and anti-Mullerian hormone (AMH) were significantly elevated (*p* < 0.001) at S1 compared to S2, and in both sites compared to RS. However, 17β-estradiol (E2) was only peaked in S1 (*p* < 0.001) compared to both S2 and RS (Figure 4).

### 3.5. Testicular Histopathology 

**Figure 5:**
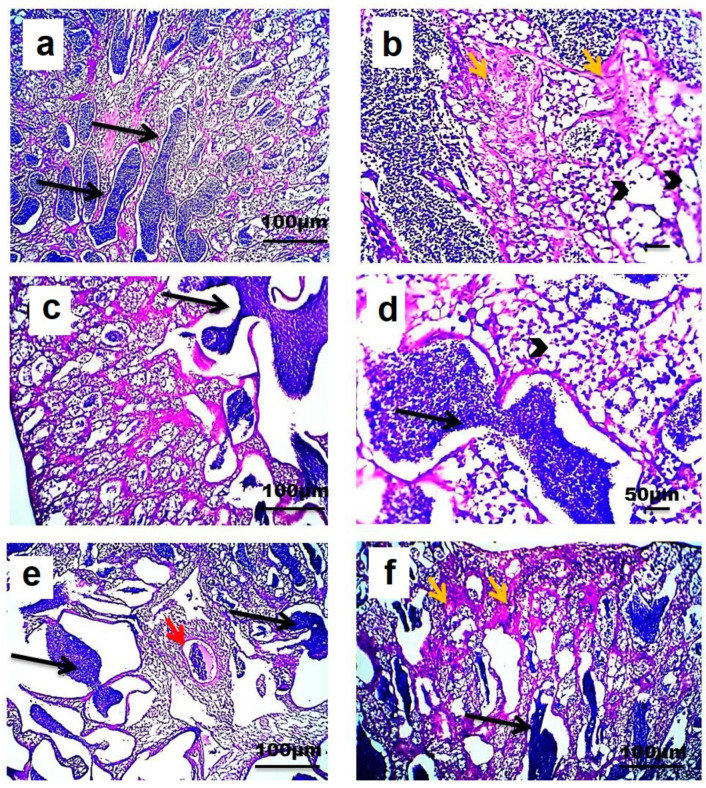
Microscopic pictures of testes of Nile tilapia from the reference site (RS): (**a**,**c**–**e**)—show accumulation of mature sperms in the lumen of the testicular lobule (black arrows) with vacuolated seminiferous epithelium (arrowheads), (**e**)—congestion (red arrow) and (**b**,**f**)—slightly increased amount of interstitial of connective tissue (orange arrows). hematoxylin and eosin (H&E), ×100: (**a**,**c**,**e**,**f**) and ×400: (**b**,**d**).

Light microscopic examination showed that longitudinally sectioned testes from the three different sites consisted of lobules separated from each other by interstitial tissue. The testes of Nile tilapia from RS consisted of lobules with cysts in different stages of development. The lobules were lined by spermatogonia, spermatocytes, and Sertoli cells. An accumulation of mature spermatozoa was observed in the lumen of testicular lobules. Vacuolated seminiferous epithelium, congestion with the presence of a melanomacrophage center, and slightly increased amount of interstitial connective tissue were also detected in testes from RS (Figure 5a–f).

**Figure 6:**
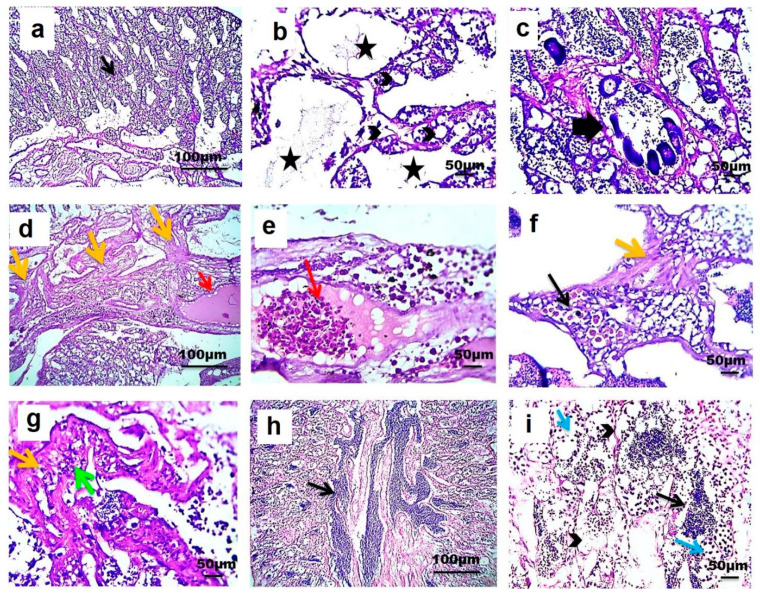
Microscopic pictures of testes from site 1 (S1): (**a**,**b**)—show vacuolization of seminiferous epithelium (arrowheads) with the absence of spermatozoa from the lumen of the testicular lobule (asterisks), (**c**)—Developmental stages of oocytes are seen inside testicular lobule (thick arrow), (**d**,**f**,**g**)—with an increased amount of interstitial of connective tissue (orange arrows); (**d**,**e**)—congestion (red arrows), (**f**)—melanomacrophage center (black arrow), (**g**)—inflammation (green arrow). Testes from site 2 (S2): (**h**,**i**)—shows a reduced number of mature sperms (black arrows) with more vacuolization of seminiferous epithelium (arrowheads) and fewer numbers of spermatogonia and spermatocytes (blue arrows). hematoxylin and eosin (H&E), ×100: (**a**,**d**,**h**) and ×400: (**b**,**c**,**e**,**f**,**g**,**i**).

However, testes of Nile tilapia from S1 showed vacuolization of the seminiferous epithelium. Considerable amounts of spermatogonia and spermatocytes were still present in tubule cysts, while spermatozoa were absent from the lumen of the testicular lobule. A few sections of the testes from this site showed the presence of oocyte developmental stages inside testicular lobule. A marked increase in interstitial connective tissue was observed alongside congestion and melanomacrophage centers and perivascular inflammation (Figure 6a–g). On another side, testes of Nile tilapia from S2 showed variation in the amount of sperm aggregation. However, mature sperms were generally reduced when compared with testis from RS. More vacuolization of the seminiferous epithelium was detected with fewer spermatogonia and spermatocytes (Figure 6h,i).

**Figure 7:**
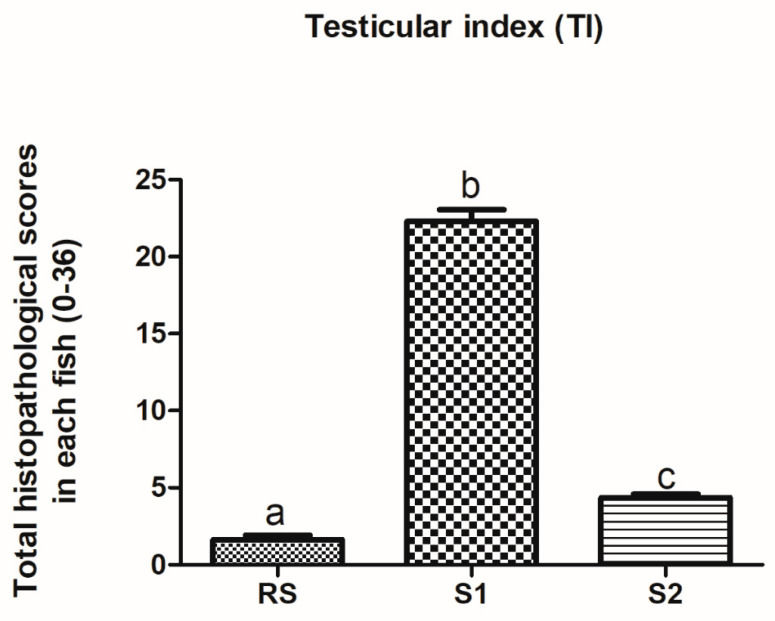
Testicular index of Nile tilapia captured from different sites. Values with a different letter superscript indicate a significant difference between sites (*p < 0.05,* Kruskal–Wallis). RS = control, S1 = site 1, S2 = site 2.

The numbers and percentage of fish affected with each testicular alteration in the three different sites were shown (Table 2). The histological response classification indicated a normal histological structure with mild histological alterations present in testes from RS (1.6 ± 0.29) and S2 (4.36 ± 0.22) regions. However, the histological response classification indicated moderate histological alterations present in testes from S1 (22.3 ± 0.74) (Figure 7).

## 4. Discussion

### 4.1. Estrogenic Endocrine Disrupting Chemicals (E-EDCs) Analysis and Biometric Measurements

E-EDCs have been associated with adverse health effects on the biological system and normal body functions of fish, resulting in alterations of the behavior pattern, reproductive function, and resistance against diseases. [34]. In the present study, testosterone, progesterone, and zeranol concentrations were detected in water sampled from different sites. However, other xenosteroids were below detectable levels. BPA was detected only at S1. Natural and synthetic estrogen-detected levels could plausibly be attributed to fertilizer application (animal manure) in farms of similar sites as these have been described as a potential source of natural hormones and veterinary pharmaceuticals [52]. In this context, zeranol, a veterinary growth promoter, suggested that this compound was released by the large animal farms nearby [52]. Natural and synthetic estrogens were only detected at low levels, possibly due to their rapid degradation in the environment, such as E1 and E2; their half-lives ranged from 2.8 to 3 days [53]. Therefore, natural steroids may be discharged at low or even undetected levels; however, they are still present in rivers at trace levels [12]. Detection of a higher level of BPA at S1, the site located to the main point of effluent source, suggested that BPA was a result of industrial activity and the WWTP effluents. BPA was not detected at S2, about 35 km from that central point of effluent source, suggesting a decreasing gradient of BPA concentration with the distance increase from the sources. This discrepancy in the BPA concentrations might be due to dilution and degradation processes. BPA concentrations started to degrade after 50 days in natural rivers [54]. Thus, BPA molecules that are not absorbed and transferred to sediment may be rapidly degraded. Overall, the detected levels of xenosteroids could vary due to several factors, such as seasonal changes, dilution factors, suspended solids, and agricultural activities [12].

In the present study, only testosterone, 17β-estradiol, and BPA were detected in the sampled fish tissues. Wastewater effluents contain mixtures of steroidal and phenolic EDCs that can interact synergistically to induce effects on the normal reproduction and development of fish at lower concentrations than those required individually. Therefore, the concentrations of steroidal EDCs in effluent-exposed fish emphasized their ability to accumulate in fish when extrapolating for possible biological effects from these compounds’ concentrations in effluents [55]. Despite being E-EDCs, responsible for levels detected at S1 and S2, the presence of the natural steroidal one at RS can be attributed to their essential role in reproduction and other physiological functions [56]. Males of teleost fish usually exhibit different levels of androgens, including testosterone as a consequence of differentiation along the male pathway [57], and they are found to have a role in efferent duct system regulation [58]. Additionally, the main biologically active estrogen in males is the estradiol, which originates from the aromatization of testosterone [59]; therefore, it is not surprising to find their levels in fish tissue from RS. 

Consistently, effluent-exposed crucian carp (*Carassius auratus*) was found to contain 4.3 ± 0.6 ng/g dw of E2 after 114 days [55]. Similarly, BPA at the level of 83.5 ng/g and 5.87 μg/g were detected in the muscle of wild fish species and from carp, respectively [60,61]. People in rural areas particularly rely on fish in their daily diet. Therefore, it is of great importance to assess the bioaccumulation of EDCs in edible fish tissues as it has implications not only for the environment but also in terms of public health [34]. BPA was the compound detected at the highest concentrations in water and fish tissues. Therefore, we hypothesized that the disruptive changes observed in the analyzed biomarkers were mostly due to BPA. 

### 4.2. Biometric and Organosomatic Indices in Fish Exposed to Estrogenic Endocrine Disrupting Chemicals (E-EDCs)

Biometric measurements, including TSI, are considered indicators of aquatic environmental pollution, such as xenosteroid [62,63]. In the current study, biometric and organosomatic measurements were significantly decreased mainly at S1 and S2 in some occasions compared to RS. The variation of the somatic growth could be due to environmental (as temperature) and microbial variations over months under consideration with a subsequent increase in effluents estrogenic potential [64]. Furthermore, these variations might be influenced by the reproductive status and general physiological condition of the fish and thereby affecting their sensitivity to E-EDCs [65]. Many studies have reported that fish growth can be reduced in polluted water [66,67]. The inhibition of gonadal growth could be attributed to xenosteroids found in the effluents discharged from the Bahr El-Baqar drain into the investigated sites. Consistently, Koi carp, *Cyprinus carpio carpio* exposed to BPA at 100 µg/L, showed decreased gonadosomatic index (GSI) with no significant differences in HSI [63]. Sohoni et al. [68] reported inhibition of both gonadal and somatic growth (weight and length) in males fathead minnows exposed to BPA concentrations of 640 and 1280 μg/L. GSI was significantly lower in mature carp naturally exposed to BPA from a highly polluted site and in *O. niloticus* exposed to 200 µg/L methomyl [62,69]. In the present study, the K-factor showed no conspicuous trend over months; however, it was declined in August and still at the same level in October. Coincided with weight and length data, K-factor changes could also be attributed to high pollutant levels [67]. As observed, HSI was noted as significantly increased at S1 and decreased at S2. HSI is considered an indicator of the metabolic load in fish and variations of HSI between S1 and S2 may be due to contaminants metabolism and increased biosynthesis caused by induction of vitellogenesis, besides in some cases toxins at low doses could display hormesis effects, which may justify non-significant changes between S1 or S2 and RS. Previous studies have reported similar results: Female and male brown trout (*Salmo trutta*) in Colorado exhibited reduced HSI in metal-contaminated sites [70]. Similarly, a previous study showed a significant increase in HSI in rainbow trout exposed to 1.5 and 15% v/v secondary treated sewage effluent for 32 weeks in flow-through mesocosms [71]. The reduced TSI and increased HSI herein could also be linked to liver hypertrophy suggesting possible effects of E-EDCs in wastewater effluent [72].

### 4.3. Endocrine Gene Expression in Fish Exposed to Estrogenic Endocrine Disrupting Chemicals (E-EDCs)

Estrogen receptors (ERs) belong to the nuclear receptor superfamily and are activated by estrogen hormone and translocated into the nucleus where they bind to target DNA and regulate gene transcription [73]. Most of the E-EDCs, including BPA, interfere with the normal estrogen-signaling pathways by interacting with three forms of ERs: *er–type I* (*er–ɑ*) and er-*type II 1* and *2 (er-ß1 and 2*). The present study represents the first report of xenosteroids effects resulting from human activities on Nile tilapia via the activation of ERs in both liver and testes. Indeed, these results are consistent with previous reports in Atlantic salmon [74], rainbow trout [75], and goldfish [76], which have shown that BPA can induce the mRNA translation for ERs proteins, suggesting that ERs, in addition to its well-established role in transcription, also modulates translation and thus govern proteome composition [77]. Furthermore, it has been shown that the estrogenic activity of BPA in fish is mediated via estrogenic receptors [78].

E-EDCs, such as BPA, are known to be more active in the ovarian cell context (CHO-K1). Furthermore, gonads have been investigated as the leading sites for the expression of *cyp19a1* [73]. In the present study, we observed for the first-time upregulation of *cyp19a1* following exposure to higher levels of BPA in Nile tilapia. The increased *cyp19a1* mRNA level could be attributed to the activation of *er-α* and *er-β* proteins in response to the increased expression of ERs in the liver and/or testes [77]. This finding was similar to the observed upregulation of *cyp 19a* mRNA levels in the gonad and the brain of *Rivulus marmoratus* and Atlantic salmon juveniles exposed to BPA and EE2. [79,80]. Further studies in zebrafish, have suggested that the increased expression of *cyp 19a1b* in glial cell model is due to activation estrogen nuclear receptor alpha (zf*er*α). In the present study, the increased expression of both ERs and *vtg* was correlated with exposure to increased *cyp 19a1* and BPA concentrations. This was also consistent with Kang et al. [81], who reported that BPA induces expression of *vtg* in male fish via its estrogenic effects.

Activation of *sox9* promotes male differentiation during early embryonic life [82]. In zebrafish, Sox9 was overexpressed in the brain and gonads but not in the liver [83]. The overexpression of *sox9* in the present study was associated with increased masculinization of fish, as reported by Shimasaki et al. [84]. However, in the present study, we observed a significant increase of *sox9* mRNA expression level in testes in S1 where E-EDCs contamination was higher than at the other sites and where the fish would be expected to be feminized by the estrogenic hormones. This conflict might be due to species variation; since *sox9* in Nile tilapia expressed intensely and equally in both types of gonads [47]. Therefore, determining the precise role of *sox9* will require more investigation. 

### 4.4. Hormones Responses in Fish Exposed to Estrogenic Endocrine Disrupting Chemicals (E-EDCs)

The hypothalamic–pituitary–gonadal axis controls the reproductive functions in mammalian species and fish. E-EDCs are found to disturb the endocrine axis signaling pathway and play a crucial role, either agonist or antagonist of their receptors [85,86]. In the present study, we investigated for the first time the effects of E-EDCs on the synthesis of gonadotropins (FSH and LH) in Nile tilapia. The present data indicated that serum FSH and LH showed the same increasing pattern at S2 than S1 and RS. These results indicated a negative feedback signaling between BPA as xenoestrogens compound and gonadotropins (LH, FSH). It was shown that exposure to BPA leads to the expression of ERs in developing hypothalamic cells [87,88]. Our findings were consistent with the observations of Qin et al. [89]. In a previous study concerning the protein expression level, BPA exposure upregulated gonadotropin-releasing hormones (GnRH) mRNA protein in zebrafish and minnow *Gobiocypris rarus* [90]. In salmon, exposure to neuroendocrine disruptors resulted in signaling changes in the GnRH system and some reproductive related genes via an ER-activated pathway [91]. The decreasing of serum LH and FSH in S1 than RS and S2 may be attributed to negative feedback signaling of serum estradiol in the same site than the other two sites as coincided with previous studies [92]. In a similar trend, decreased serum estradiol level in S2 was associated with increased LH and FSH levels. Low serum estrogens concentrations may have a significant stimulatory effect on the secretion and synthesis of gonadotropins [71].

The expression of AMH inhibits the development of the paramesonephric duct [93]. As observed, the serum level of AMH was significantly higher at S1 than S2 and RS. Additionally, serum estradiol and testosterone levels were found to have the same AMH pattern, suggesting that higher serum AMH is a consequence of increased serum testosterone in fish from the same site. Further, higher serum gonadotropins and Vtg in trout were associated with higher serum estradiol concentration [71].

### 4.5. Testicular Histopathology in Fish Exposed to Estrogenic Endocrine Disrupting Chemicals (E-EDCs)

The histopathological alterations in the fish testes from S1 showed more lesions than encountered in testes from S2 and RS. The most striking feature was the absence of mature spermatozoa from the lumen of the testicular lobule; besides, developmental stages of oocytes inside testicular lobule were seen in a few testicular sections from S1 only. Although the percentage prevalence of testicular oocytes was relatively low (13.3%), it is regarded as a severe and irreversible condition. In most cases, the intersex seemed to be the result of the feminization of male specimens as testicular tissue was predominant, with single or groups of oocytes present throughout the seminiferous tissue.

In a similar fashion, BPA induced detrimental effects on gonadal tissue, decreasing spermatozoa, and the loss of testicular structure with abnormal testicular connective tissue in male Japanese medaka *Oryzias latipes* [94,95]. While a higher BPA exposure of 10 μg/L was found to cause testes degeneration with tumefaction of the sustentacular cells and a decrease in spermatozoa, this compound similarly inhibits spermatogenesis in fathead minnow *Pimephales promelas* [68]. Consistent with our observation, cases of intersex state (testis–ova) were seen in testicular tissue of male Nile tilapia, suggesting that Vtg induction and hormonal disruption in male fish are associated with the appearance of intersex state [96]. Our findings are consistent with Mandich et al. [97], where spermatogenesis inhibition was evident. Additionally, BPA exposure at level 16 μg/L in fathead minnows elicited a decrease in the number of mature spermatozoa [68]. Lower concentrations of BPA (1 and 10 μg/L) did not significantly affect the cell population percentages; however, increasing the exposure dose can decrease the numbers of mature shapes [98]. These observations reported suggesting that BPA causes modulations in sperm maturation and could result in adverse effects on sperm quality [63].

## 5. Conclusions

In summary, levels of E-EDCs were detected in water and fish tissues sampled from three farms nearby and subjected to fill from the Bahr El-Baqar drain. Inhibition of fish biometric and organosomatic indices, disruption of hormonal balance, and gene expression regulation were evident and associated with the levels of E-EDCs contamination, mainly BPA, as it was the dominating compound and the maximum concentration detected. Significant histopathological alterations in fish testes confirmed our findings. To the best of our knowledge, the present study represents the first comprehensive study evaluating the adverse effects of E-EDCs from effluents on fish health in Egypt. Our findings give important insights into these negative impacts not only on the ecosystem but also on human health. Therefore, immediate strategies for controlling pollution in aquaculture should be developed; for example, aquaculture could be allowed to use good quality water rather than being only allowed to use water from agricultural drainage and reused water sources as per Egyptian law. Thus, achieving fish welfare and maintain aquaculture sustainability. 

## Figures and Tables

**Table 1:** Steroidal and phenolic endocrine disruptive chemicals (EDCs) in water (µg/L) and fish tissues (µg/kg) from sampling sites along the Bahr El-Baqar drainage (Egypt).

	Water Samples			Tissue Samples								
Target Chemical	RS	S1	S2	Testes			Muscle			Liver		
				RS	S1	S2	RS	S1	S2	RS	S1	S2
**Natural Hormones (µg/L)**				**Natural Hormones (µg/kg)**								
Estrone (E1)	<LOQ	<LOQ	<LOQ	<LOQ	<LOQ	<LOQ	<LOQ	<LOQ	<LOQ	<LOQ	<LOQ	<LOQ
17β–Estradiol (E2)	<LOQ	<LOQ	<LOQ	1.91	<LOQ	5.01	<LOQ	1.05	<LOQ	<LOQ	<LOQ	<LOQ
Hexsterol	<LOQ	<LOQ	<LOQ	<LOQ	<LOQ	<LOQ	<LOQ	<LOQ	<LOQ	<LOQ	<LOQ	<LOQ
Diethyl stilbesterol	<LOQ	<LOQ	<LOQ	<LOQ	<LOQ	<LOQ	<LOQ	<LOQ	<LOQ	<LOQ	<LOQ	<LOQ
Dienesterol	<LOQ	<LOQ	<LOQ	<LOQ	<LOQ	<LOQ	<LOQ	<LOQ	<LOQ	<LOQ	<LOQ	<LOQ
Testosterone	0.12	0.2	<LOQ	0.16	6	1.1	0.19	0.23	0.57	0.68	0.41	3.8
Progesterone	0.21	0.13	<LOQ	<LOQ	<LOQ	<LOQ	<LOQ	<LOQ	<LOQ	<LOQ	<LOQ	<LOQ
**Veterinary Pharmaceuticals (µg L^−1^)**				**Veterinary Pharmaceuticals (µg kg^−1^)**								
Zeranol	0.51	<LOQ	3.44	<LOQ	<LOQ	<LOQ	<LOQ	<LOQ	<LOQ	<LOQ	<LOQ	<LOQ
**Industrial/Domestic Compound (µg/mL)**				**Industrial/Domestic Compound (µg/kg)**								
Bisphenol A	ND	6.5	ND				ND	25.9	ND	ND	48.07	ND

ND = not detected; RS = reference site; S1 = Site 1; S2 = Site 2; LOQ = Limit of quantification.

**Table 2:** Percentage prevalence of the histological alterations identified in the testes of Nile tilapia from sampling sites.

Site of Sample Collection	Reaction Pattern	Histopathological Examination	Frequency (*n* = 60)	%
RS	RC	vacuolated seminiferous epithelium	10	16.6
	CD	congestion	12	20
	RC	Melano-macrophage centers	10	16.6
S1	RC	Vacuolization of the seminiferous epithelium	35	58.3
	RC	No spermatozoa in the lumen of the testicular lobule	18	30
	RC	Melano-macrophage centers	25	41.6
	PC	Wall proliferation of lobular cysts	25	41.6
	I	Mononuclear leukocytes	33	55
	CD	congestion	42	70
	IS	Testicular oocytes	8	13.3
S2	RC	Vacuolization of the seminiferous epithelium	15	25
	CD	congestion	10	16.6

RS = reference site, S1 = Site 1, and S2 = Site 2. CD = circulatory disturbances, RC = retrogressive changes; PC = progressive change, I = inflammation, and IS = intersex (IS).

## Appendix A

**Table A1:** Primers used for quantitative real-time RT-PCR.

Gene Name	GenBank No.	Description	Annealing Temperature (°C)	Sequence (5′–3′)
*er-type I*	NM_001279770.1	Forward Reverse	55	AGCGACAAATCAGTGCACCA ATTGCACGCTTCCTTCCATC
*Er-type II*	NM_001279774.1	Forward Reverse	55	AGCATCCAAGGACACAACGA ATGCCCTTTGGTTCAGCTGT
*cyp19a1*	NM_001279586.1	Forward	54	TCAAACAAAACCCGCACGTG
		Reverse		AAAACTCGGTGCGGTGCATT
*sox9*	XM_003450119.4	Forward Reverse	56	CGGGAGAGCATTCAGGTCAGTC CAGCTTTGCTGGAGGGAAGG
*vtg*	FJ709597	Forward Reverse	59	AGACCCTCAGTTGCTGGAGT CGGTGTCGAGAGCTGAGTAG
*β-actin*	EU887951.1	Forward Reverse	60	GTTGCCATCCAGGCTGTGCT TCTCGGCTGTGGTGGTGAAG

## References

[1] General Authority for Fish Resources Development GAFRD (2014). Fish Statistics Year Book.

[2] El-Sayed A.-F., Dickson M., El-Naggar G.O. (2015). Value chain analysis of the aquaculture feed sector in Egypt. Aquaculture.

[3] El-Sayed A.F.M. (2017). Regional Review on Status and Trends in Aquaculture Development in the Near East and North Africa-2015.

[4] Ojaveer H., Tomkiewicz J., Arula T., Klais R. (2015). Female ovarian abnormalities and reproductive failure of autumn-spawning herring (*Clupea harengus membras*) in the Baltic Sea. ICES J. Mar. Sci..

[5] Molnar J.L., Gamboa R.L., Revenga C., Spalding M.D. (2008). Assessing the global threat of invasive species to marine biodiversity. Front. Ecol. Environ..

[6] Peña-Mendoza B., Gómez-Márquez J.L., Salgado-Ugarte I.H., Ramirez-Noguera D. (2007). Reproductive biology of Oreochromis niloticus (*Perciformes: Cichlidae*) at Emiliano Zapata dam, Morelos, Mexico. Rev. Biol. Trop..

[7] Swaminathan M. (2012). Aquaculture and sustainable nutrition security in a warming planet. J. Farming Waters People.

[8] Soliman N.F. (2017). Aquaculture in Egypt under Changing Climate.

[9] Shaalan M., El-Mahdy M., Saleh M., El-Matbouli M. (2017). Aquaculture in Egypt: Insights on the current trends and future perspectives for sustainable development. Rev. Fish. Sci. Aquac..

[10] Muliari M., Akmal Y., Zulfahmi I., Karja N.W., Nisa C., Mahyana M., Humairani R. (2020). Effect of exposure to palm oil mill effluent on reproductive impairment of male Nile Tilapia (*Oreochromis niloticus, Linnaeus 1758*). E3S Web Conf..

[11] Scholz S., Klüver N. (2009). Effects of endocrine disrupters on sexual, gonadal development in fish. Sex. Dev..

[12] Jeffries K.M., Jackson L.J., Ikonomou M.G., Habibi H.R. (2010). Presence of natural and anthropogenic organic contaminants and potential fish health impacts along two river gradients in Alberta, Canada. Environ. Toxicol. Chem..

[13] Peng X., Yu Y., Tang C., Tan J., Huang Q., Wang Z. (2008). Occurrence of steroid estrogens, endocrine-disrupting phenols, and acid pharmaceutical residues in urban riverine water of the Pearl River Delta, South China. Sci. Total Environ..

[14] Blazer V.S., Iwanowicz L.R., Henderson H., Mazik P.M., Jenkins J., Alvarez D.A., Young J.A. (2011). Reproductive endocrine disruption in smallmouth bass (*Micropterus dolomieu*) in the Potomac River basin: Spatial and temporal comparisons of biological effects. Environ. Monit. Assess..

[15] Hu J., Zhang Y., Zhou R. (2009). Changes in extracellular osmolality initiate sperm motility in freshwater teleost rosy barb Puntius conchonius. Theriogenology.

[16] Desbrow C., Routledge E., Brighty G.C., Sumpter J.P., Waldock M. (1998). Identification of estrogenic chemicals in STW effluent. 1. Chemical fractionation and In Vitro biological screening. Environ. Sci. Technol..

[17] Wright-Walters M., Volz C. (2009). Municipal wastewater concentrations of pharmaceutical and xeno-estrogens: Wildlife and human health implications. Proceedings of the 2007 National Conference on Environmental Science and Technology.

[18] Palanza P., Nagel S.C., Parmigiani S., vom Saal F.S. (2016). Perinatal exposure to endocrine disruptors: Sex, timing and behavioral endpoints. Curr. Opin. Behav. Sci..

[19] Xu N., Xu Y.-F., Xu S., Li J., Tao H.-C. (2012). Removal of estrogens in municipal wastewater treatment plants: A Chinese perspective. Environ. Pollut..

[20] Behera S.K., Kim H.W., Oh J.-E., Park H. (2011). Occurrence and removal of antibiotics, hormones and several other pharmaceuticals in wastewater treatment plants of the largest industrial city of Korea. Sci. Total Environ..

[21] Quednow K., Püttmann W. (2008). Endocrine disruptors in freshwater streams of Hesse, Germany: Changes in concentration levels in the time span from 2003 to 2005. Environ. Pollut..

[22] Wee S.Y., Aris A.Z. (2017). Endocrine disrupting compounds in drinking water supply system and human health risk implication. Environ. Int..

[23] Zheng W., Li X., Yates S.R., Bradford S.A. (2012). Anaerobic transformation kinetics and mechanism of steroid estrogenic hormones in dairy lagoon water. Environ. Sci. Technol..

[24] Bergé A., Gasperi J., Rocher V., Gras L., Coursimault A., Moilleron R. (2014). Phthalates and alkylphenols in industrial and domestic effluents: Case of Paris conurbation (France). Sci. Total Environ..

[25] Richardson S.D., Ternes T.A. (2014). Water analysis: Emerging contaminants and current issues. Anal. Chem..

[26] Oehlmann J., Schulte-Oehlmann U., Breure A.M., Markert B.A., Zechmeister H.G. (2002). Molluscs as bioindicators. Trace Metals and Other Contaminants in the Environment.

[27] Frye C., Bo E., Calamandrei G., Calza L., Dessì-Fulgheri F., Fernández M., Fusani L., Kah O., Kajta M., Le Page Y. (2011). Endocrine disrupters: A review of some sources, effects, and mechanisms of actions on behaviour and neuroendocrine systems. J. Neuroendocr..

[28] Gu Y., Yu J., Hu X., Yin D. (2016). Characteristics of the alkylphenol and bisphenol A distributions in marine organisms and implications for human health: A case study of the East China Sea. Sci. Total Environ..

[29] Lee C.-C., Jiang L.-Y., Kuo Y.-L., Hsieh C.-Y., Chen C.S., Tien C.-J. (2013). The potential role of water quality parameters on occurrence of nonylphenol and bisphenol A and identification of their discharge sources in the river ecosystems. Chemosphere.

[30] Shao B., Han H., Hu J., Zhao J., Wu G., Xue Y., Ma Y., Zhang S. (2005). Determination of alkylphenol and bisphenol A in beverages using liquid chromatography/electrospray ionization tandem mass spectrometry. Anal. Chim. Acta.

[31] Wei X., Huang Y., Wong M.H., Giesy J.P., Wong C.K.C. (2011). Assessment of risk to humans of bisphenol A in marine and freshwater fish from Pearl River Delta, China. Chemosphere.

[32] Villeneuve D.L., Garcia-Reyero N., Escalon B.L., Jensen K.M., Cavallin J.E., Makynen E.A., Durhan E.J., Kahl M.D., Thomas L.M., Perkins E.J. (2011). Ecotoxicogenomics to support ecological risk assessment: A case study with bisphenol A in fish. Environ. Sci. Technol..

[33] Diamanti-Kandarakis E., Bourguignon J.-P., Giudice L.C., Hauser R., Prins G.S., Soto A.M., Zoeller R.T., Gore A.C. (2009). Endocrine-disrupting chemicals: An Endocrine Society scientific statement. Endocr. Rev..

[34] Xu Z., Liu J., Gu L.-P., Huang B., Pan X. (2017). Biological effects of xenoestrogens and the functional mechanisms via genomic and nongenomic pathways. Environ. Rev..

[35] Meng S., Qiu L., Hu G., Fan L., Song C., Zheng Y., Wu W., Qu J., Li D., Chen J. (2016). Effects of methomyl on steroidogenic gene transcription of the hypothalamic-pituitary-gonad-liver axis in male tilapia. Chemosphere.

[36] Filby A.L., Thorpe K.L., Maack G., Tyler C. (2007). gene expression profiles revealing the mechanisms of anti-androgen and estrogen-induced feminization in fish. Aquat. Toxicol..

[37] Liu S., Chen H., Xu X.-R., Liu S.-S., Sun K.-F., Zhao J.-L., Ying G.-G. (2015). Steroids in marine aquaculture farms surrounding Hailing Island, South China: Occurrence, bioconcentration, and human dietary exposure. Sci. Total Environ..

[38] Rasmussen E.K., Petersen O.S., Thompson J.R., Flower R.J., Ahmed M.H. (2009). Hydrodynamic-ecological model analyses of the water quality of Lake Manzala (Nile Delta, Northern Egypt). Hydrobiologia.

[39] Ghanem A., Haggag M. (2015). Assessment of the feasibility of using filter made of rice straw for treating aquaculture effluents in Egypt. Resour. Environ..

[40] Saad A. (1997). Environmental Hydrogeologic Impacts of Ground Water Withdrawal in the Eastern Nile Delta Region with Emphasis on Ground-Water Pollution Potential. Ph.D. Thesis.

[41] Monem A.M.A., Gharieb M.M., Hussian A.-E.M., Flefil N.S. (2017). Sensitivity of phytolankton to the wastewater quality discharging at lake Manzala, Egypt. J. Int. J. Ocean..

[42] Taha A., El-Mahmoudi A., El-Haddad I. (2004). Pollution sources and related environmental impacts in the new communities Southeast Nile Delta, Egypt. J. Emir. J. Eng. Res..

[43] Elbana T.A., Bakr N., Karajeh F., El Quosy F. (2014). Treated Wastewater Utilization for Agricultural Irrigation in Egypt. The National Conference on Water Quality: Challenges and Solutions.

[44] Zavala M.A.L., Arriaga B.N.F., Funamizu N. (2016). Simultaneous determination of four estrogens in compost based on ultrasonic solvent extraction, solid-phase extraction clean-up and analysis by UHPLC-MS/MS. Am. J. Anal. Chem..

[45] Xiao Q., Li Y., Ouyang H., Xu P., Wu D. (2006). High-performance liquid chromatographic analysis of bisphenol A and 4-nonylphenol in serum, liver and testis tissues after oral administration to rats and its application to toxicokinetic study. J. Chromatogr. B.

[46] Livak K.J., Schmittgen T.D. (2001). Analysis of relative gene expression data using real-time quantitative Pcr and the 2^−ΔΔ*C*_T_^ method. J. Methods.

[47] Ijiri S., Kaneko H., Kobayashi T., Wang D., Sakai F., Paul-Prasanth B., Nakamura M., Nagahama Y. (2008). Sexual dimorphic expression of genes in gonads during early differentiation of a teleost fish, the Nile Tilapia *Oreochromis niloticus*. Boil. Reprod..

[48] Abo-Al-Ela H.G., El-Nahas A., Mahmoud S., Ibrahim E.M. (2017). The extent to which immunity, apoptosis and detoxification gene expression interact with 17 alpha-methyltestosterone. Fish Shellfish. Immunol..

[49] Kevenaar M.E., Meerasahib M.F., Kramer P., Van De Lang-Born B.M.N., De Jong F.H., Groome N.P., Themmen A.P.N., Visser J.A. (2006). Serum anti-müllerian hormone levels reflect the size of the primordial follicle pool in mice. Endocrinology.

[50] Bancroft J.D., Gamble M. (2008). Theory and Practice of Histological Techniques.

[51] Zimmerli S., Bernet D., Burkhardt-Holm P., Schmidt-Posthaus H., Vonlanthen P., Wahli T., Segner H. (2007). Assessment of fish health status in four Swiss rivers showing a decline of brown trout catches. Aquat. Sci..

[52] Kolok A.S., Snow D.D., Kohno S., Sellin M.K., Guillette L.J. (2007). Occurrence and biological effect of exogenous steroids in the Elkhorn River, Nebraska, USA. Sci. Total Environ..

[53] Bowman J.C., Readman J.W., Zhou J.L. (2003). Sorption of the natural endocrine disruptors, oestrone and 17β-oestradiol in the aquatic environment. Environ. Geochem. Health.

[54] Kocaman E., Ozhan K. (2019). Degradation of bisphenol A in natural and artificial marine and freshwaters in Turkey. Bull. Environ. Contam. Toxicol..

[55] Liu J., Wang R., Huang B., Lin C., Zhou J., Pan X. (2012). Biological effects and bioaccumulation of steroidal and phenolic endocrine disrupting chemicals in high-back crucian carp exposed to wastewater treatment plant effluents. Environ. Pollut..

[56] Phumyu N., Boonanuntanasarn S., Jangprai A., Yoshizaki G., Na-Nakorn U. (2012). Pubertal effects of 17α-methyltestosterone on GH–IGF-related genes of the hypothalamic–pituitary–liver–gonadal axis and other biological parameters in male, female and sex-reversed Nile tilapia. Gen. Comp. Endocrinol..

[57] Fernandino J.I., Hattori R.S., Kishii A., Strüssmann C.A., Somoza G.M. (2012). The cortisol and androgen pathways cross talk in high temperature-induced masculinization: The 11β-hydroxysteroid dehydrogenase as a key enzyme. Endocrinology.

[58] De Waal P.P., Wang D.S., Nijenhuis W.A., Schulz R., Bogerd J. (2008). Functional characterization and expression analysis of the androgen receptor in zebrafish (*Danio rerio*) testis. Reproduction.

[59] Leavy M., Trottmann M., Liedl B., Reese S., Stief C., Freitag B., Baugh J., Spagnoli G., Kölle S. (2017). Effects of elevated β-estradiol levels on the functional morphology of the testis—New insights. Sci. Rep..

[60] Liu J., Wang R., Huang B., Lin C., Wang Y., Pan X. (2011). Distribution and bioaccumulation of steroidal and phenolic endocrine disrupting chemicals in wild fish species from Dianchi Lake, China. Environ. Pollut..

[61] Mortazavi S., Bakhtiari A.R., Esmaili-Sari A., Bahramifar N., Rahbarizadeh F. (2013). Occurrence of endocrine disruption chemicals (bisphenol A, 4-nonylphenol, and octylphenol) in muscle and liver of *Cyprinus Carpino* common from Anzali Wetland, Iran. Bull. Environ. Contam. Toxicol..

[62] Hassanin A., Kuwahara S., Nurhidayat, Tsukamoto Y., Ogawa K., Hiramatsu K., Sasaki F. (2002). Gonadosomatic index and testis morphology of common carp (*Cyprinus carpio*) in rivers contaminated with estrogenic chemicals. J. Vet. Med. Sci..

[63] Al-Sakran A.A.M., Virk P., Elobeid M., Hamed S.S., Siddiqui M.I., Omer S., Mirghani N.M. (2016). Histopathological effects on testis of adult male carp, *Cyprinus carpio carpio*, following exposure to graded concentrations of water-borne bisphenol A. Trop. J. Pharm. Res..

[64] Rodgers-Gray T.P., Jobling S., Morris S., Kelly C., Kirby S., Janbakhsh A., Harries J.E., Waldock M.J., Sumpter J.P., Tyler C.R. (2000). Long-term temporal changes in the estrogenic composition of treated sewage effluent and its biological effects on fish. Environ. Sci. Technol..

[65] Hemming J.M., Allen H., Thuesen K.A., Turner P.K., Waller W.T., Lazorchak J.M., Lattier D., Chow M., Denslow N., Venables B. (2004). Temporal and spatial variability in the estrogenicity of a municipal wastewater effluent. Ecotoxicol. Environ. Saf..

[66] Bakhoum S., Faltas S. (1994). The influence of water pollution upon the growth performance of *Oreqchromis Aureus (Steinlj.*) in lake Mariut, Egypt. Bull. Nat. Inst. Oceanogr. Fish..

[67] Khallaf E.A., Galal M., Authman M.M. (2003). The biology of *Oreochromis niloticus* in a polluted canal. Ecotoxicology.

[68] Sohoni P., Tyler C.R., Hurd K., Caunter J., Hetheridge M., Williams T., Woods C., Evans M., Toy R., Gargas M. (2001). Reproductive effects of long-term exposure to Bisphenol A in the fathead minnow (*Pimephales promelas*). Environ. Sci. Technol..

[69] Meng S., Qiu L., Hu G., Fan L., Song C., Zheng Y., Wu W., Qu J., Li D., Chen J. (2017). Effect of methomyl on sex steroid hormone and vitellogenin levels in serum of male tilapia (*Oreochromis niloticus*) and recovery pattern. Environ. Toxicol..

[70] Norris D.O., Camp J.M., Maldonado T.A., Woodling J.D. (2000). Some aspects of hepatic function in feral brown trout, *Salmo trutta*, living in metal contaminated water. Comp. Biochem. Physiol. Part C Pharmacol. Toxicol. Endocrinol..

[71] Höger B., Taylor S., Hitzfeld B., Dietrich D., van den Heuvel M.P. (2006). Stimulation of reproductive growth in rainbow trout (*Oncorhynchus mykiss*) following exposure to treated sewage effluent. Environ. Toxicol. Chem..

[72] Hemming J.M., Waller W.T., Chow M.C., Denslow N.D., Venables B. (2001). Assessment of the estrogenicity and toxicity of a domestic wastewater effluent flowing through a constructed wetland system using biomarkers in male fathead minnows (*pimephales promelas Rafinesque*). Environ. Toxicol. Chem..

[73] Tokarz J., Möller G., De Angelis M.H., Adamski J. (2015). Steroids in teleost fishes: A functional point of view. Steroids.

[74] Yadetie F., Arukwe A., Goksøyr A., Male R. (1999). Induction of hepatic estrogen receptor in juvenile Atlantic salmon In Vivo by the environmental estrogen, 4-nonylphenol. Sci. Total Environ..

[75] Arukwe A. (2001). Differential biomarker gene and protein expressions in nonylphenol and estradiol-17β treated juvenile rainbow trout (*Oncorhynchus mykiss*). Comp. Biochem. Physiol. Part C Toxicol. Pharmacol..

[76] Soverchia L., Ruggeri B., Palermo F.A., Mosconi G., Cardinaletti G., Scortichini G., Gatti G., Polzonettimagni A. (2005). Modulation of vitellogenin synthesis through estrogen receptor beta-1 in goldfish (*Carassius Auratus*) juveniles exposed to 17-β estradiol and nonylphenol. Toxicol. Appl. Pharmacol..

[77] Bronson M.W., Hillenmeyer S., Park R.W., Brodsky A.S. (2010). Estrogen coordinates translation and transcription, revealing a role for NRSF in human breast cancer cells. Mol. Endocrinol..

[78] Gibert Y., Sassi-Messai S., Fini J.-B., Bernard L., Zalko D., Cravedi J.-P., Balaguer P., Andersson-Lendahl M., Demeneix B.A., Laudet V. (2011). Bisphenol A induces otolith malformations during vertebrate embryogenesis. BMC Dev. Boil..

[79] Lee Y.-M., Seo J.S., Kim I.-C., Yoon Y.-D., Lee J.-S. (2006). Endocrine disrupting chemicals (bisphenol A, 4-nonylphenol, 4-tert-octylphenol) modulate expression of two distinct cytochrome P450 aromatase genes differently in gender types of the hermaphroditic fish *Rivulus marmoratus*. Biochem. Biophys. Res. Commun..

[80] Lyssimachou A., Jenssen B.M., Arukwe A. (2006). Brain cytochrome P450 Aromatase gene isoforms and activity levels in Atlantic salmon after waterborne exposure to nominal environmental concentrations of the pharmaceutical ethynylestradiol and antifoulant tributyltin. Toxicol. Sci..

[81] Kang J.-H., Aasi D., Katayama Y. (2007). Bisphenol A in the aquatic environment and its endocrine-disruptive effects on aquatic organisms. Crit. Rev. Toxicol..

[82] Cui J., Shen X., Zhao H., Nagahama Y. (2011). Genome-wide analysis of sox genes in Medaka (*Oryzias latipes*) and their expression pattern in embryonic development. Cytogenet. Genome Res..

[83] Caballero-Gallardo K., Olivero-Verbel J., Freeman J.L. (2016). Toxicogenomics to evaluate endocrine disrupting effects of environmental chemicals using the zebrafish model. Curr. Genom..

[84] Shimasaki Y., Kitano T., Oshima Y., Inoue S., Imada N., Honjo T. (2003). Tributyltin causes masculinization in fish. Environ. Toxicol. Chem..

[85] Zhang J., Zuo Z., Chen R., Chen Y., Wang C. (2008). Tributyltin exposure causes brain damage in *Sebastiscus marmoratus*. Chemosphere.

[86] Vandenberg L.N., Maffini M.V., Sonnenschein C., Rubin B.S., Soto A.M. (2008). Bisphenol-A and the great divide: A review of controversies in the field of endocrine disruption. Endocr. Rev..

[87] Wang H., Wang J., Wu T., Qin F., Hu X., Wang L., Wang Z. (2011). Molecular characterization of estrogen receptor genes in *Gobiocypris rarus* and their expression upon endocrine disrupting chemicals exposure in juveniles. Aquat. Toxicol..

[88] Yang M., Qiu W., Chen B., Chen J., Liu S., Wu M., Wang K.-J. (2015). The In Vitro immune modulatory effect of bisphenol A on fish macrophages via estrogen receptor A and nuclear factor-Κb signaling. J. Environ. Sci. Technol..

[89] Qin F., Wang L., Wang X., Liu S., Xu P., Wang H., Wu T., Zhang Y., Zheng Y., Li M. (2013). Bisphenol A affects gene expression of gonadotropin-releasing hormones and type I GnRH receptors in brains of adult rare minnow *Gobiocypris rarus*. Comp. Biochem. Physiol. Part C Toxicol. Pharmacol..

[90] Vosges M., Le Page Y., Chung B.-C., Combarnous Y., Porcher J., Kah O., Brion F. (2010). 17α-Ethinylestradiol disrupts the ontogeny of the forebrain GnRH system and the expression of brain aromatase during early development of zebrafish. Aquat. Toxicol..

[91] Harding L.B., Schultz I.R., Goetz G.W., Luckenbach J.A., Young G., Goetz F.W., Swanson P. (2013). High-throughput sequencing and pathway analysis reveal alteration of the pituitary transcriptome by 17α-ethynylestradiol (EE2) in female coho salmon, *Oncorhynchus kisutch*. Aquat. Toxicol..

[92] Pasmanik M., Schlinger B.A., Callard G.V. (1988). In Vivo steroid regulation of aromatase and 5α-reductase in goldfish brain and pituitary. Gen. Comp. Endocrinol..

[93] Behringer R.R. (1994). 5 the In Vivo Roles of müllerian-inhibiting substance. Current Topics in Developmental Biology.

[94] Yokota H., Tsuruda Y., Maeda M., Oshima Y., Tadokoro H., Nakazono A., Honjo T., Kobayashi K. (2000). Effect of bisphenol A on the early life stage in Japanese Medaka (*Oryzias Latipes*). Environ. Toxicol. Chem..

[95] Metcalfe D.C., Metcalfe T.L., Kiparissis Y., Koenig B.G., Khan C., Hughes R.J., Croley T.R., March E.R., Potter T. (2001). Estrogenic potency of chemicals detected in sewage treatment plant effluents as determined by In Vivo assays with Japanese Medaka (*Oryzias Latipes*). J. Environ. Toxicol. Chem. Biodivers..

[96] Ali T., Abdel-Aziz S., El-Sayed A.-F., Zeid S. (2017). Effects of nonylphenol on plasma steroids, vitellogenin synthesis and sex reversal in Nile Tilapia (*Oreochromis Niloticus*) fingerlings. Indian J. Geo Mar. Sci..

[97] Mandich A., Bottero S., Benfenati E., Cevasco A., Erratico C., Maggioni S., Massari A., Pedemonte F., Vigano L. (2007). In Vivo exposure of carp to graded concentrations of bisphenol A. Gen. Comp. Endocrinol..

[98] Lora A., Molina A., Bellido C., Blanco A., Monterde J. (2016). Moyano Adverse effects of bisphenol A on the testicular parenchyma of zebrafish revealed using histomorphological methods. J. Veterinární Med..

